# Hfq-Antisense RNA I Binding Regulates RNase E-Dependent RNA Stability and ColE1 Plasmid Copy Number

**DOI:** 10.3390/ijms25073955

**Published:** 2024-04-02

**Authors:** Wei-Syuan Wang, Sue Lin-Chao

**Affiliations:** 1 Molecular and Cell Biology, Taiwan International Graduate Program, Academia Sinica and Graduate Institute of Life Science, National Defense Medical Center, Taipei 11490, Taiwan; 2 Institute of Molecular Medicine, College of Medicine, National Taiwan University, Taipei 10002, Taiwan; 3 Institute of Molecular Biology, Academia Sinica, Taipei 11529, Taiwan

**Keywords:** antisense RNA, *E. coli*, pUC plasmid, RNase E, RNA stability, RNA-binding protein, sRNA competition

## Abstract

The mechanisms and consequences of gene regulation by Hfq on *trans*-encoded small RNAs (sRNAs) have been well studied and documented. Recent employment of Genomic SELEX to search for Hfq-binding motifs has indicated that Hfq might frequently regulate gene expression controlled by *cis*-antisense RNAs. Here, we use the classic ColE1 plasmid antisense RNA-based regulation model (i.e., RNA I) to study the role of Hfq in controlling antisense regulatory functions. We show that Hfq exhibits a high binding affinity for RNA I and that binding limits RNase E cleavage, thereby stabilizing RNA I and reducing the plasmid copy number. Full-length RNA I displays a binding affinity for Hfq in the sub-micromolar range. In vivo overexpression of Hfq prolongs RNA I stability and reduces the ColE1 plasmid copy number, whereas deletion of *hfq* reduces RNA I stability and increases the plasmid copy number. RNA I predominantly binds to the proximal face of Hfq and exhibits competitive ability against a chromosome-borne proximal face-bound sRNA (DsrA) for Hfq binding. Through its strong promoter and high gene dosage features, plasmid-encoded antisense RNA I results in high RNA I expression, so it may antagonize the effects of *trans*-encoded RNAs in controlling target gene expression.

## 1. Introduction

Bacteria have developed numerous strategies to regulate gene expression, including through small regulatory RNAs (sRNAs), which can be classified as either *trans*-encoded or *cis*-encoded sRNAs (or antisense RNAs) [1]. *Trans*-encoded sRNAs are encoded elsewhere on the chromosome from their target mRNAs and, hence, display imperfect base-pairing. In contrast, antisense RNAs are perfectly complementary to their respective sense target RNAs since they are encoded on the same genetic locus, albeit on the opposite strand to their regulatory target. The base-pairing interaction of *trans*-encoded sRNAs and their target mRNAs is frequently dependent on RNA chaperones (e.g., Hfq) [2]. At the molecular level, *trans*-encoded sRNAs regulate gene expression of the target mRNAs by forming RNA–RNA base-pairing through a short segment of typically 7 to 12 base pairs (bps), known as the seed region [3]. However, the base-paired stretch can vary from 6 bps (in the case of SgrS-*ptsG* [4]) to >30 bps (in the case of Spot42-*galK* [5]). Hfq-mediated sRNA–mRNA base-pairing can activate the RNA targets [6] or induce their degradation [7]. Blueprints of many Hfq-dependent sRNA regulatory networks can be established beyond the known targetomes using genome-wide approaches, such as RNA interaction by ligation and sequencing (RIL-seq), global sRNA target identification by ligation and sequencing (GRIL-Seq), and UV crosslinking, ligation, and sequencing of hybrids (CLASH) [8,9,10,11].

Hfq (originally identified as a host factor required for replication of bacteriophage Qβ RNA [12,13]) is one of the most extensively studied RNA chaperones in recent decades. It is a member of the Sm/LSm family that forms homo-hexameric doughnut-like structures in bacteria [14]. Structural studies of the Hfq–RNA complex have revealed that the proximal face of Hfq preferentially binds to U-rich single-stranded RNA [15], whereas the distal face of Hfq preferentially binds to A-rich extensions [16]. Binding by the two Hfq surfaces brings RNA species into close proximity for further sRNA–mRNA base-pairing. The arginine patch on the outer rim is responsible for the chaperone activity required for RNA annealing [17]. Mutations in this arginine patch do not prevent RNA binding to either the proximal or distal face [17]. The Hfq C-terminal domain can self-assemble into amyloid-like fibrillar structures and may displace RNA from Hfq [18,19,20]. Coincidently, Hfq’s binding preference for A/U-rich sequences is similar to the targeting sequence of ribonuclease E (RNase E, i.e., single-stranded AU-rich sequences), and several sRNA-based negative regulatory mechanisms are known to be involved in RNase E-mediated RNA degradation [7,21,22].

Studies using Hfq coimmunoprecipitation (coIP) with deep-sequencing analyses have uncovered several Hfq-bound antisense RNAs [23,24]. Furthermore, genomic SELEX (Systematic Evolution of Ligands by EXponential enrichment) has been applied to identify Hfq-binding motifs (Hfq aptamers), which revealed that the vast majority of Hfq aptamers mapped to antisense strands opposite to the translation initiation sites and in the intervening sequences between open reading frames (ORFs) in operons, indicating a role for Hfq in *cis*-antisense RNA regulation [25]. As Hfq is extensively involved in *trans*-encoded sRNA regulation, only a few studies have investigated its role in antisense RNA regulation of target translation [26,27], and it is generally accepted that Hfq may not be required for pairing of most of the antisense strand with the fully complementary sense RNA strand. More studies are needed to understand antisense RNA–Hfq binding and its effects on the control of diverse biological functions.

**Figure 1:**
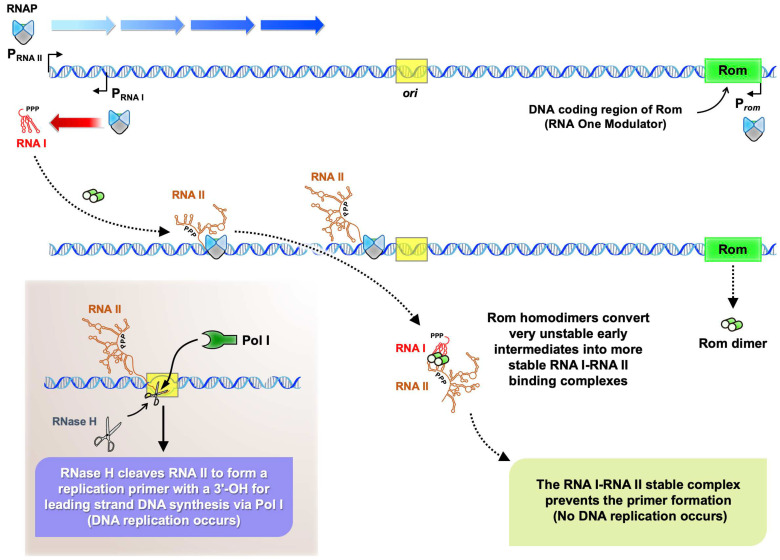
ColE1 plasmid DNA replication is controlled by antisense RNA–RNA interactions. The diagram shows the molecular mechanism of plasmid-borne RNA I, RNA II, and the Rom RNA-binding protein in controlling ColE1 plasmid replication (based on Tomizawa and co-authors [34,35,38,40]). The RNA I transcript of ColE1, termed antisense RNA, inhibits plasmid DNA replication. RNA I interacts dynamically with the pre-primer RNA (RNA II), with the Rom protein binding to both RNAs and converting the unstable RNA I–RNA II transient intermediate into a more stable RNA I–RNA II complex, shown as dashed lines from top to bottom, thereby preventing primer formation and inhibiting DNA replication. In contrast, in the absence of RNA I binding (bottom left), the alternative structure of RNA II forms a RNA–DNA hybrid near the replication origin and undergoes RNase H cleavage, producing a functional replication primer with a 3′ hydroxyl group for DNA polymerase I to synthesize leading-strand DNA. The promoter orientation of each transcript is labeled, with the RNA polymerase and DNA polymerase I shown as RNAP and Pol I, respectively. The RNA One Modulator is also labeled as Rom. The *ori* of ColE1 is also indicated.

Most naturally occurring antisense RNA-regulated systems have been characterized in bacterial plasmids or mobile genetic elements (e.g., transposons) [28]. The first natural antisense RNAs were discovered by studying control of plasmid DNA replication [29,30]. Replication of the ColE1 plasmid (one of the most frequently used plasmids in modern molecular cloning studies) is one of the classic RNA-based regulation models. ColE1 replication is tightly controlled by the plasmid-borne RNA primer, RNA II, its antisense regulator, i.e., RNA I [31,32], and it requires host proteins, including RNA polymerase, RNase H, and DNA polymerase I [33,34]. Transcription of RNA II starts from 555 bp upstream of the DNA replication origin, and the nascent RNA II has two alternative conformational structures [35]. One conformation of the RNA II pre-primer is able to form an RNA–DNA hybrid with the plasmid DNA near the replication origin [36,37], is cleaved by RNase H, and generates a 3′ hydroxyl group for DNA polymerase I-catalyzed synthesis of the DNA leading strand [33]. However, the RNA II primer conformation is inhibited by the antisense RNA I (encoded 445 bp upstream of the replication origin, and its synthesis proceeds in the opposite direction to that of RNA II [32]), which alters the secondary structure of RNA II and thus prevents primer formation [35,38]. The RNA I–RNA II complex initiates via transient interaction of single-stranded loop regions of each RNA to form the so-called “kissing complex” [39]. The kissing complex is further stabilized by a plasmid-encoded 63-amino acid (aa) protein, Rom (RNA One Modulator) [40]. The stable RNA I–RNA II complex inhibits primer formation for DNA replication [35,38,39]. In Figure 1, we present a schematic of the antisense RNA regulatory mechanism of ColE1 replication.

The stability of antisense RNA I is considered a critical factor in regulating the ColE1 plasmid copy number [41,42]. RNA I degradation starts with a rate-limiting step of RNase E-mediated cleavage between the fifth and sixth nucleotides of its single-stranded AU-rich 5′ end and it is followed by rapid degradation with the aid of PAP I-mediated 3′ polyadenylation [41,42,43,44]. The ColE1-like plasmid copy number is also believed to be affected by Hfq, as indicated by the [^3^H]thymidine incorporation rate increasing relative to wildtype upon *hfq* deletion [45]. Additionally, deploying a synchrotron radiation-based circular dichroism (SRCD) approach on the amyloid-like C-terminal domain of Hfq (residues 64 to 102), the first stem-loop of the RNA I fragment (5′ ACA GUA UUU GGU AUC UGC GCU CUG CUG AAG CCA GUU ACC 3′), and its complementary stem-loop primer fragment, revealed that this Hfq C-terminal polypeptide could aid in annealing both RNA fragments [46].

Since the rate of processing and degradation of antisense RNA I regulates the replication of ColE1-type plasmids [41], Hfq-driven regulation of antisense RNA stability and the plasmid copy number control requires further assessment. In this study, we have combined in vitro and in vivo approaches to determine how Hfq binds to RNA I, the Hfq-binding face of RNA I, and the impact of Hfq on RNase E-mediated RNA I degradation, which thereby affects the ColE1 plasmid copy number.

## 2. Results

### 2.1. Deletion of the RNA I 5′ End Single-Stranded Region Significantly Reduces Hfq–RNA I Binding, and High-Affinity Hfq Binding Limits RNase E Cleavage

**Figure 2:**
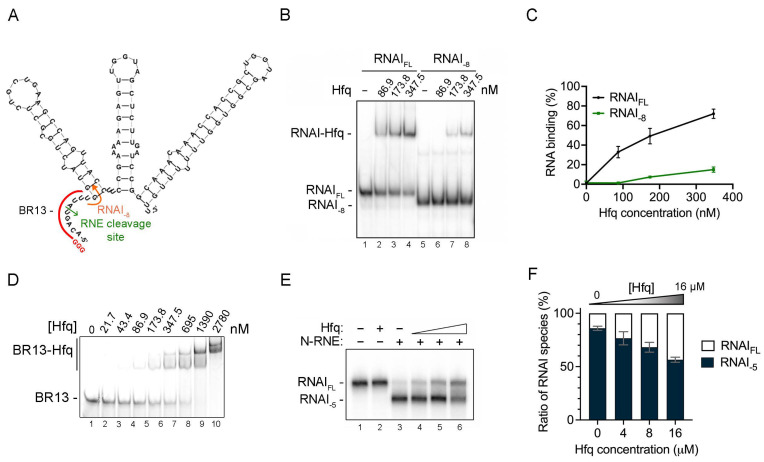
Hfq exhibits high-affinity binding to the single-stranded 5′ end of RNA I, and Hfq binding limits RNase E cleavage. (**A**) Structure and nucleotide sequence of RNA I. The region of BR13 is marked with a red line. The RNase E cleavage site is marked by a green arrowhead. The RNA I_-8_ variant encoded from the ninth nucleotide of RNA I is marked in orange. (**B**) In vitro gel-shift assay. Free-form RNA I variants (labeled RNA I_FL_ and RNA I_-8_) and Hfq-bound RNA I variants (labeled RNA I–Hfq) are indicated. Hfq monomer concentrations (nM) in the binding reaction are indicated at the top of the gel. Samples were resolved by 6% native PAGE. (**C**) Quantification of the binding percentage of RNA I against Hfq concentration. Free-form RNA I signal intensity in the absence of Hfq protein (lanes 1 and 5, respectively) was considered as 0% binding. Free-form RNA I signal in the presence of Hfq protein was used to calculate the percentage of binding relative to 0 nM Hfq. Quantification was based on three technical repeats. (**D**) In vitro gel-shift assay. BR13 and its Hfq-associated complex are labeled. Samples were resolved by 8% native PAGE. (**E**) In vitro RNase E cleavage assay in the absence or presence of Hfq. Full-length RNA I (RNA I_FL_) and the RNase E cleavage product, RNA I_-5_, are indicated. Reactions lacking Hfq and/or N-RNE protein are indicated at the top of the gel. Monomer Hfq concentrations of 0, 4, 8, or 16 μM and an N-RNE concentration of 750 nM were used. Samples were resolved on an 8% polyacrylamide (19:1)/7 M urea gel. (**F**) Quantification (based on three technical repeats) of the ratio between the full-length RNA I signal (RNA I_FL_, white bar) and the N-RNE cleavage product (RNA I_-5_, black). The Y-axis represents the cleavage result based on the percentages of the un-cleaved (RNA I) and cleaved (RNA I_-5_) RNA I species. The X-axis indicates the Hfq monomer concentration.

The rate-limiting step of RNA I degradation is RNase E cleavage between the 5th and 6th nucleotide of its 5′ end AU-rich single-stranded region [41,44]. RNase E is involved in the negative regulation of many sRNAs [7], and it has been shown that the AU-rich single-stranded sequences of many sRNAs are coincidently preferred for both Hfq binding and RNase E cleavage [21]. However, whether Hfq can bind to the 5′ end AU-rich region of RNA I and affect RNase E cleavage is unclear. Here, we synthesized in vitro full-length RNA I (RNA I_FL_, sequence shown in Figure 2A) and a 5′ end single-stranded deletion variant (RNA I_-8_, marked in Figure 2A), which lacks the first eight nucleotides containing the 5′ end AU-rich RNase E cleavage site (namely 5′-ACA GUA UU-3′), for an Hfq–RNA binding experiment (described in the Section 4 Materials and Methods). As shown in Figure 2B, RNA I_FL_ began to display a band shift signal at a monomeric Hfq concentration of 86.9 nM (Figure 2B, lane 2), reflecting formation of an RNA I–Hfq complex. In contrast, RNA I_-8_ failed to form a complex with Hfq protein under the same condition (Figure 2B, lane 6). Quantification analysis revealed that 72.1 ± 6.8% (relative to the total intensity signal) of RNA I_FL_ formed complexes at an Hfq monomer concentration of 347.5 nM (Figure 2C, black line), whereas only 15.0 ± 2.7% of RNA I_-8_ formed Hfq-RNA complexes (Figure 2C, green line). The in vitro gel-shift assay with single-stranded BR13 (the sequence of which is identical to the first 13 mers of T7-synthesized RNA I, marked in red in Figure 2A) also revealed binding of Hfq, as indicated by the band-shift signal (Figure 2D). Although we cannot rule out that other stem-loop regions of RNA I act as putative Hfq binding sites, our data indicate that deletion of the single-stranded AU-rich region at the 5′ end of RNA I significantly reduces the formation of Hfq–RNA complexes and that the 5′ end single-stranded oligonucleotide of RNA I is sufficient for Hfq binding.

Next, we explored if Hfq binding affects RNase E cleavage at the RNA I 5′ end. Hfq is known to interact with the C-terminal region of RNase E (residues 711 to 750) [47]. To circumvent any bias or modulation introduced by Hfq-C-terminal RNase E protein–protein interactions, we used a purified C-terminal-truncated RNase E variant (N-RNE). The N-RNE polypeptide (residues 1 to 597) retains the RNase E enzymatic domain. RNase E cleavage was assessed in the absence or presence of Hfq. In the absence of Hfq protein, 86% of RNA I was cleaved by N-RNE in our in vitro RNase E cleavage assay, yielding RNA I_-5_ (Figure 2E, lane 3; Figure 2F). However, as the amounts of Hfq protein increased (from 4 to 16 µM Hfq monomer), we detected more RNA I as remaining intact (Figure 2E, lanes 4–6; Figure 2F). In the presence of 16 µM Hfq, 43.5 ± 2.3% of RNA I was protected from N-RNE cleavage (Figure 2E, lane 6; Figure 2F, white bar in column 4), which means that 56.5% RNA I_-5_ was generated. As Hfq is able to form Hfq–RNA I complex at a nanomolar concentration (shown in Figure 2B), this incomplete protection by Hfq at a micromolar concentration (16 µM) indicates that RNA I possess other Hfq binding sites (discussed further below). Nevertheless, our data show that the 5′ end single-stranded AU-rich region of RNA I coincidently serves for both RNase E cleavage and Hfq binding.

### 2.2. RNA I Displays a Sub-Micromolar Binding Affinity for Hfq Protein In Vitro

**Figure 3:**
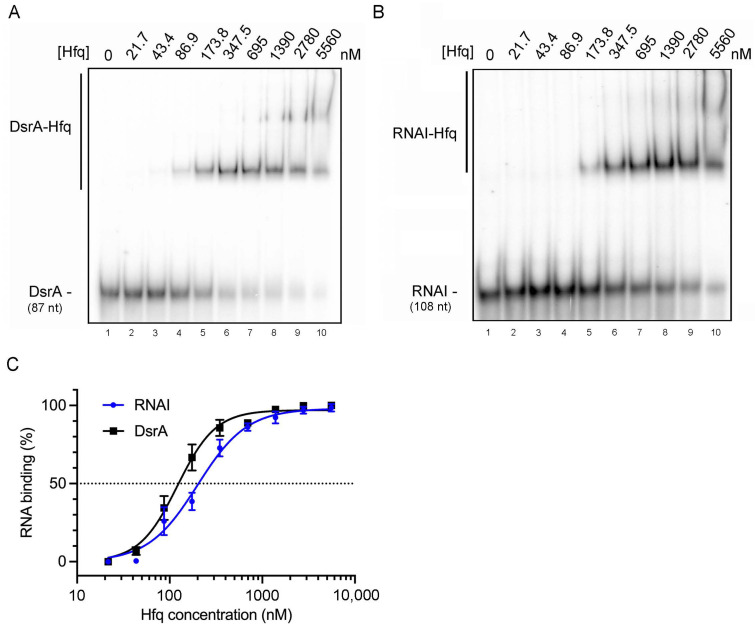
RNA I displays a sub-micromolar binding affinity for Hfq protein in vitro. (**A**) Gel-shift mobility assay of DsrA–Hfq interactions. Free-form (DsrA) and the Hfq-bound form (DsrA-Hfq) are as indicated. The Hfq monomer concentration is shown at the top of the gel. Samples were resolved by 6% native PAGE. (**B**) Gel-shift mobility assay of RNA I–Hfq interactions. (**C**) Quantification of RNA–Hfq binding percentages. The Y-axis shows the percentage of Hfq-bound RNA relative to total RNA, setting lane 1 as 0% binding. The X-axis indicates the Hfq monomer concentration (nM) in log scale. Quantification was based on at least three technical repeats.

Next, we determined the binding affinity of Hfq for RNA I. Hfq–RNA binding affinities were determined by the gel-shift assay, as described in the Section 4 Materials and Methods. Here, we compared the binding affinity of RNA I to DsrA (87 nucleotides), one of the earliest identified and well-characterized sRNAs that can bind Hfq [48]. The single-stranded AU-rich region between stem loops I and II of DsrA primarily serves as an Hfq binding site [49], with both the proximal face and the outer rim of Hfq interacting with DsrA, as mutations within these surfaces reduce the respective amounts of DsrA co-immunoprecipitated with Hfq and its steady-state levels [50]. Our in vitro gel-shift assay on DsrA and Hfq complexes revealed that the first band-shift appeared at a monomeric Hfq concentration of 86.9 nM (Figure 3A, lane 4), with a second band-shift arising at an Hfq concentration of 695 nM (Figure 3A, lane 7). Quantification showed that the first DsrA binding affinity (K_1_) was 122 ± 12 nM Hfq monomer (Figure 3C). The binding affinities of DsrA reported in the literature are 221 nM Hfq monomer for K_1_ and 4 μM Hfq monomer for K_2_ [51], with another group reporting 21 ± 1 nM Hfq_6_ for DsrA K_1_ and 94 ± 5 nM Hfq_6_ for K_2_ (i.e., about 126 nM Hfq monomer and 564 nM Hfq monomer, respectively) [52]. Thus, our binding affinity data were consistent with previous studies.

Under the same reaction conditions, we determined the binding affinity of RNA I for Hfq. Similar to our results for DsrA, RNA I also displayed two gel-shift signals in the in vitro gel-shift assay. The first band-shift appeared at a monomeric Hfq concentration of 173.8 nM (Figure 3B lane 5), and a second band-shift appeared at an Hfq concentration of 1390 nM (Figure 3B lane 8). Quantification revealed the first RNA I binding affinity (K_1_) to be 225 ± 21 nM Hfq monomer (Figure 3C). Thus, RNA I has a lower binding affinity for Hfq compared to DsrA, yet our results demonstrated that RNA I exhibits a binding affinity for Hfq in the sub-micromolar range that is comparable to previously reported binding affinities for other sRNAs [51,53].

### 2.3. Proximal Face Mutants of Hfq Decrease Both DsrA and RNA I Binding, and These Two RNAs Compete with Each Other for Hfq Binding

**Figure 4:**
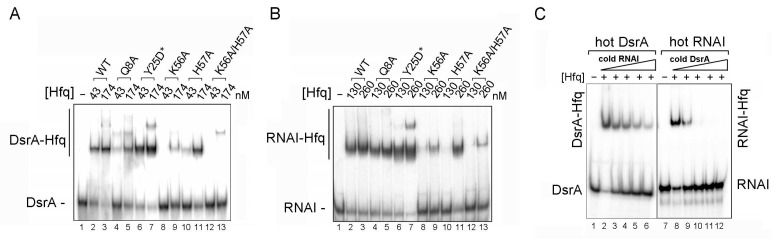
The proximal, but not distal, face of Hfq mediates binding of both RNA I and DsrA, and the two RNA species compete with each other for Hfq binding. (**A**) Gel-shift mobility assay of DsrA with Hfq mutant variants. Free-form RNA (DsrA) and the Hfq-bound form (DsrA-Hfq) are indicated. Hfq variants and their monomer concentrations (nM) are indicated at the top of the gel. Samples were resolved by 6% native PAGE. (**B**) In vitro gel-shift mobility assay of RNA I with Hfq mutant variants. (**C**) Competition assay between DsrA and RNA I. Free-form RNA (labeled as either DsrA or RNA I) and the respective Hfq-bound forms (labeled as DsrA–Hfq or RNA I–Hfq) are indicated. ^32^P-labeled RNA (labeled as either hot DsrA or hot RNA I) and non-isotope-labeled RNA (labeled as either cold DsrA or cold RNA I) are as indicated at the top of the gel. Reactions lacking Hfq monomer are indicated. Increasing concentrations of cold DsrA, ranging from 25 to 200 nM, and cold RNA I, ranging from 850 nM to 6.8 μM, are represented by white triangles. Competition reactions were resolved by 6% native PAGE.

Hfq has three RNA-binding surfaces [2], i.e., the proximal face that preferentially binds single-stranded U-rich sequences [15], a distal face that preferentially binds A-rich sequences [16], and a rim that helps RNA–RNA annealing with low binding sequence specificity [17]. Class I sRNAs bind to the proximal face and the outer rim of Hfq, whereas class II sRNAs interact with both the proximal and distal faces of Hfq protein [2]. We determined which Hfq face (proximal or/and distal face) interacts with RNA I using Hfq proximal face (Q8A, K56A, H57A, and K56A/H57A) and distal face (Y25D*) mutants, following Mikulecky et al. [52]. We used DsrA as a control to validate the effects of Hfq mutations on Hfq–sRNA binding. Based on the binding affinity results shown in Figure 3A, we selected 43 nM and 174 nM Hfq monomer concentrations to analyze binding of DsrA to wildtype or mutant Hfq (Figure 4). In the gel-shift assays, we observed that the proximal face mutants (Q8A, K56A, H57A, and K56A/H57A) disrupted DsrA binding to a greater extent than wildtype (with the highest disruption identified for the K56A/H57A double-mutant, binding of 13.2 ± 6.9% relative to binding of wildtype Hfq, Hfq_WT_), as evidenced by more free-form RNA appearing at the bottom of the gel (Figure 4A, and relative binding percentage shown in Table 1). In contrast, the distal face mutation (Y25D*) slightly enhanced DsrA binding (Figure 4A, lanes 6 and 7) relative to Hfq_WT_ (Figure 4A, lanes 2 and 3). This higher binding to the Y25D* mutant is consistent with a previous report by Mikulecky et al., showing that the distal face Y25D* mutant has a higher binding affinity for DsrA (K_d_ of 16 nM of Hfq_Y25D*_ hexamer, i.e., 96 nM of Hfq_Y25D*_ monomer, compared to the K_d_ of 21 nM of Hfq_WT_ hexamer, i.e., 126 nM of Hfq_WT_ monomer) [52].

Using these same Hfq mutants under identical reaction conditions, we analyzed the surface by which RNA I binds to Hfq. Since RNA I has a lower binding affinity for Hfq than DsrA, for this experiment we used 130 nM and 260 nM Hfq monomer concentrations. Whereas the percentage binding of RNA I was reduced to varying degrees for proximal face mutants (also with the highest decrease observed for the K56A/H57A double-mutant, with 13.8 ± 8.6% relative to binding of Hfq_WT_; Figure 4B and Table 1), the distal face mutant slightly increased the RNA I binding percentage (Figure 4B, lanes 6 and 7), indicating that like DsrA, RNA I interacts with the same face of Hfq.

To validate this result that RNA I and DsrA interact with the same Hfq surface, we used in vitro competition experiments to determine if these two RNAs compete with each other for Hfq binding in the presence of a limited amount of Hfq protein. We used non-isotope-labeled (cold) RNA as a competitor against an isotopically labeled (hot) RNA species. Although DsrA exhibits a higher binding affinity for Hfq than RNA I, we observed that by increasing the amount of cold RNA I (from 850 ng to 6.8 μg), it gradually outcompeted DsrA for Hfq binding, as revealed by more free-form (unbound) DsrA signal detected in the bottom of the gel (Figure 4C, left panel, lanes 2–6). Likewise, cold DsrA could outcompete hot RNA I for binding of Hfq (Figure 4C, right panel, lanes 8–12), albeit at a lower cold RNA concentration (from 25 ng to 200 ng), consistent with its higher binding affinity for Hfq. Taken together, these data demonstrate that plasmid-borne RNA I can bind to the proximal face of Hfq with a high binding affinity and is able to compete with a chromosome-borne sRNA (DsrA) for Hfq binding in vitro.

### 2.4. Hfq protects RNA I from RNase E-Mediated Degradation Predominantly through Its Proximal but Not Distal Face in E. coli

**Figure 5:**
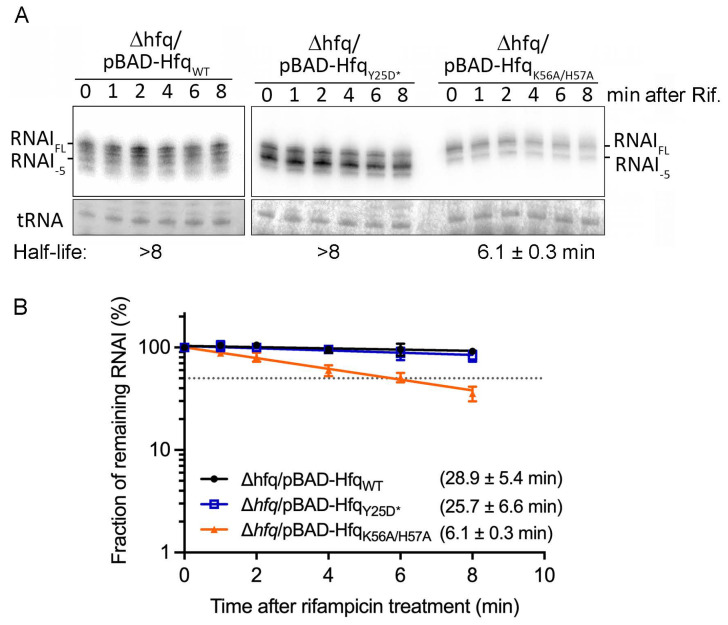
Hfq protects RNA I from RNase E-mediated degradation primarily through its proximal face. (**A**) Northern blot of RNA I. Full-length RNA I (RNA I_FL_) and its degradation product (RNA I_-5_) are indicated. Methylene blue staining of tRNA was used as a loading control. Time after rifampicin treatment and the strains from which total RNA were isolated are shown. Average half-lives were calculated from at least three biological repeats. (**B**) RNA I degradation rate. The Y-axis indicates the signals of remnant RNA I from each lane after rifampicin treatment compared to time 0. Signal from time 0 was set as 100%. Fraction (%) of remnant RNA I is plotted in log scale on the Y-axis. The X-axis indicates the time before and after rifampicin treatment (min). Δ*hfq*/pBAD-Hfq_WT_ is shown in black, Δ*hfq*/pBAD-Hfq_Y25D*_ in blue, and Δ*hfq*/pBAD-Hfq_K56A/H57A_ in orange. Half-life was determined as described in the Section 4 Materials and Methods.

As shown in Figure 2, Hfq binding protects RNA I from RNase E cleavage in vitro. Moreover, RNA I binds Hfq through its proximal, but not distal, face (Figure 4). Since RNase E cleavage at the 5′ end of RNA I is the rate-limiting step for RNA I degradation, we explored if Hfq binding could stabilize RNA I in vivo and whether the resulting stabilization effect preferentially operates through the Hfq proximal face. To do so, we ectopically expressed Hfq_WT_, a proximal face double-mutant (Hfq_K56A/H57A_), or a distal face mutant (Hfq_Y25D*_) in *Escherichia coli* lacking the chromosomally encoded *hfq* gene (i.e., *hfq* deletion), and then measured RNA I stability to compare how these mutations affect RNA I stability (i.e., protection of RNA I from degradation). We employed rifampicin to stop de novo RNA synthesis and then determined the amounts of RNA remaining at different time points after rifampicin treatment. RNA I stability for the Hfq_Y25D*_ distal face mutant (Δ*hfq*/pBAD-Hfq_Y25D*_) was equivalent to that for the Hfq_WT_ strain (Δ*hfq*/pBAD-Hfq_WT_; half-life >8 min, calculated as 25.7 ± 6.6 min and 28.9 ± 5.4 min, respectively; Figure 5A,B), indicating that the distal face mutation (Y25D*) does not impair the ability of Hfq to protect RNA I from RNase E cleavage. Notably, the Hfq_WT_-expressing strain generated more RNA I_FL_ than the Hfq_Y25D*_-expressing strain, which accumulated more RNA I_-5_ (Figure 5A), as discussed further below. In contrast to the distal face mutant, the Hfq proximal face double-mutant (Δ*hfq*/pBAD-Hfq_K56A/H57A_) significantly shortened the RNA I half-life from >25 min to 6.1 ± 0.3 min (Figure 5A,B), supporting that mutations in the proximal face limit Hfq from adequately protecting RNA I from degradation. Therefore, the protective ability of Hfq for RNA I predominantly operates through its proximal face.

### 2.5. Hfq Controls ColE1 Plasmid Copy Number by Regulating RNA I Stability

**Figure 6:**
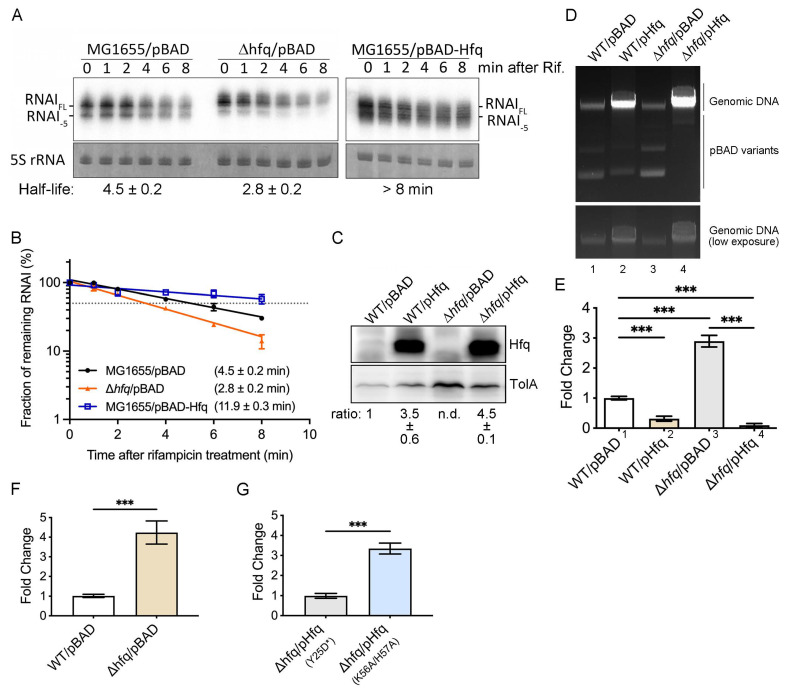
Hfq controls the ColE1-type plasmid copy number by regulating RNA I stability. (**A**) Northern blot of RNA I. Methylene blue staining of 5S rRNA was used as a loading control. Labels are as described in Figure 5A. (**B**) RNA I degradation rate. Labels are as described in Figure 5B. MG1655/pBAD is shown in black, Δ*hfq*/pBAD in orange, and MG1655/pBAD-Hfq in blue. (**C**) Western blot of Hfq protein. TolA was used as a loading control. The Hfq expression ratio was obtained from three biological repeats by setting the Hfq level in the MG1655/pBAD strain as 1. (**D**) DNA gel showing relative amounts of ColE1 plasmid. Genomic DNA is indicated and is used for normalization. A low-exposure image of the genomic DNA signal is shown in the bottom panel. Plasmid DNA in its monomeric and dimeric forms is labeled as pBAD variants. Samples were resolved on a 0.6% agarose gel. (**E**) Relative fold-change of the plasmid copy number among the different strains. Genomic DNA signal intensities from low-exposure film were used to normalize the plasmid intensities of each strain. Normalized plasmid intensity from MG1655/pBAD was set as 1 to compare the relative fold-changes in plasmid intensity displayed by the other strains. The Y-axis indicates the fold-changes in plasmid signal intensity, with sample type plotted on the X-axis. *** *p*-value < 0.001, from an unpaired Student’s *t* test. (**F**) Relative fold-change of the plasmid copy number among the different strains by qPCR. Plasmid-borne gene *ampA* was normalized against chromosome-borne gene *mreB* to obtain the ΔCt of each strain. MG1655/pBAD was set as 1 to compare the relative fold-change of plasmid level in the Δ*hfq*/pBAD strain. The Y-axis indicates the fold-changes (2^−ΔΔCt^) in plasmid level, with sample type plotted on the X-axis. *** *p*-value < 0.001, from an unpaired Student’s *t* test. (**G**) Relative fold-change of plasmid copy number among the different strains by qPCR. Description as above, except Δ*hfq*/pBAD-Hfq_Y25D*_ was set as 1 to compare the relative fold-change of the plasmid level in the Δ*hfq*/pBAD-Hfq_K56A/H57A_ strain.

Finally, we assessed if Hfq-mediated protection of RNA I could reduce the plasmid copy number by determining if RNA I stability is correlated with the plasmid copy number in the MG1655 strain and an isogenic *hfq* deletion strain. In the presence of endogenous Hfq, the RNA I half-life in the MG1655/pBAD strain was 4.5 ± 0.2 min, whereas in the Δ*hfq*/pBAD strain it was 2.8 ± 0.2 min (Figure 6A,B). Hfq gain-of-function in the wildtype strain (MG1655/pBAD-Hfq) prolonged the RNA I half-life to 11.9 ± 0.3 min (Figure 6A,B). Although both MG1655/pBAD-Hfq (Figure 6B) and Δ*hfq*/pBAD-Hfq_WT_ (Figure 5B) exhibited prolonged RNA I half-lives, the RNA I half-life was more stable in Δ*hfq*/pBAD-Hfq (28.9 ± 5.4 min) than in MG1655/pBAD-Hfq (11.9 ± 0.3 min). Western blot analysis revealed greater Hfq protein expression in Δ*hfq*/pBAD-Hfq than in MG1655/pBAD-Hfq (~4.5-fold and ~3.5-fold increases, respectively, Figure 6C). Although ectopically expressed Hfq was induced from the same plasmid (pBAD-Hfq_WT_) and with the same final concentration of 0.2% arabinose, Hfq induction somehow differed slightly, resulting in the RNA I half-life being prolonged to different extents in the two tested strains. Nevertheless, these data indicate that the amount of Hfq expressed in vivo is positively correlated with the stability of RNA I, i.e., RNA I is more stable at higher Hfq levels.

Next, we determined plasmid copy numbers in these strains, with each sample quantitatively normalized according to its chromosomal DNA. As shown in Figure 6, in the absence of Hfq, Δ*hfq*/pBAD (Figure 6D,E, lane 3) produced approximately three times more plasmid DNA than the wildtype strain MG1655/pBAD (Figure 6D,E, lane 1). In contrast, overexpression of Hfq in MG1655 (MG1655/pBAD-Hfq; Figure 6D,E, lane 2), or in an *hfq* deletion strain (Δ*hfq*/pBAD-Hfq; Figure 6D,E, lane 4), resulted in a significant reduction in the plasmid copy number compared to wildtype, with plasmid copy numbers being reduced to 0.3-fold or 0.1-fold (*p*-value < 0.001, from an unpaired Student’s *t* test), respectively, that of wildtype. The higher signal intensity at the chromosome level in MG1655/pBAD-Hfq or Δ*hfq*/pBAD-Hfq (Figure 6D, lane 2 or 4) is due to higher DNA loading for quantitative purposes, as described in the Section 4 Materials and Methods. This outcome is consistent with the rapid RNA I degradation observed in the absence of Hfq (Δ*hfq*/pBAD) and its greater stability in the presence of Hfq (MG1655/pBAD-Hfq and Δ*hfq*/pBAD-Hfq), as shown in Figure 5A and Figure 6A. Additionally, RNA I proved more stable in the Δ*hfq*/pBAD-Hfq than MG1655/pBAD-Hfq strain (Figure 5B and Figure 6B, respectively), with the plasmid copy number in Δ*hfq*/pBAD-Hfq, therefore, being correspondingly lower (Figure 6D,E, lane 4 compared to lane 2).

As shown in Figure 4, RNA I predominantly binds to the proximal, but not distal, face of Hfq, and in Figure 5 we show that Hfq proximal face mutations influence RNA I stability more than a distal face mutation, relative to wildtype. We further explored if Hfq proximal face mutations have a greater functional impact on the plasmid copy number than the distal face mutation. By using qPCR, first, we evidenced reproducibility of the results from Figure 6D,E, showing ~4.2-fold more plasmid DNA normalized with chromosome DNA in Δ*hfq*/pBAD than in MG1655/pBAD (Figure 6F), supporting the feasibility of the qPCR approach. Next, by comparing the relative plasmid copy number in the proximal face double-mutant overexpression strain (Δ*hfq*/pBAD-Hfq_K56A/H57A_) and distal face mutant overexpression strain (Δ*hfq*/pBAD-Hfq_Y25D*_), we revealed that the proximal face K56A/H57A double-mutant exhibited a ~3.3-fold higher plasmid level than the distal face Y25D* mutant strain (Figure 6G). These data support that the Hfq proximal face binds to RNA I, stabilizes it, and hence negatively controls the ColE1 plasmid copy number in vivo.

## 3. Discussion

Hfq is a functionally robust protein that regulates gene expression primarily at the post-transcriptional level by binding and facilitating RNA–RNA base-pairing [2]. Although numerous studies have examined the role of Hfq in regulating chromosome-borne *trans*-encoded sRNAs, few have assessed how it is involved in regulating antisense RNAs.

Antisense RNA-mediated regulation of plasmid replication is the classic RNA-based regulation model. Plasmids are important circular DNA molecules extensively used in both basic research and biotechnology. The ColE1 plasmid and its derivatives are some of the most commonly adapted plasmids for cloning. DNA replication of the ColE1 plasmid is negatively regulated by the antisense RNA, RNA I, the base pairs of which are perfectly complimentary to the 5′ end of its sense strand RNA pre-primer, RNA II, thereby inhibiting formation of the alternative RNA II secondary structure for primer formation (Figure 1) [32,38]. The ColE1 plasmid expresses higher levels (5-fold) of RNA I than RNA II, enabling tight control of the plasmid copy number in bacterial cells [54]. However, the RNA I–RNA II interaction intermediate is unstable, with the plasmid-borne small protein Rom assisting in converting the transient intermediate into the stable RNA I–RNA II complex (Figure 1) [40]. Deletion of the *rom* gene from ColE1-derived plasmids in order to obtain higher yields of plasmid DNA molecules is, therefore, commonly used in the fields of molecular engineering and DNA technology (such as to produce DNA vaccines). The high yields of plasmid DNA molecules further increases RNA I abundance in the host bacteria. Considering the high RNA I abundance produced from high-copy-number ColE1-derived plasmids and the important role played by the RNA chaperone Hfq, establishing how antisense RNA I interacts with Hfq and through which binding surface, as well as the consequences of this interaction, are of general interest.

In our study, we used full-length RNA I and intact Hfq protein to perform in vitro binding and competition assays, as well as a combination of genetic approaches, to show that RNA I predominantly interacts with the proximal, but not distal, face of Hfq. This interaction enhances the stability of RNA I, thereby reducing the ColE1 plasmid copy number. The stability of RNA I is known to be a critical factor in regulating the ColE1 plasmid copy number [41,42], and we have shown herein that by prolonging RNA I stability, Hfq is correlated with a reduced ColE1 copy number. Nevertheless, it remains to be established if Hfq functions similar to Rom to facilitate RNA I–RNA II base-pairing in *E. coli*.

### 3.1. Hfq Might Have Other Binding Sites in Addition to the 5′ end Single-Stranded AU-Rich Region of RNA I

Many sRNAs are stabilized by binding of Hfq since both Hfq and RNase E share similar target sequences (i.e., AU-rich single-stranded sequences) for either binding or cleavage [21]. ColE1 plasmid-borne antisense RNA I is a well-known target of RNase E that is cleaved between the fifth and sixth nucleotides of its 5′ end single-stranded AU-rich region [43,44]. Our in vitro Hfq binding and protection of RNase E cleavage assay demonstrated binding of Hfq to that region of RNA I, thereby reducing RNase E cleavage, and our in vivo RNA I half-life data showed that Hfq expression prevents RNA I from degradation (Figure 2 and Figure 6). However, despite using a high-micromolar concentration of Hfq (16 μM monomer, which proved sufficient to form the Hfq–RNA I complex, Figure 3B) in our N-RNE cleavage assay (Figure 2E, lane 6), it still could not completely protect RNA I_FL_ from N-RNE cleavage (at an N-RNE concentration of 750 nM; Figure 2E, lane 6). The intracellular Hfq level in *E. coli* is ~55,000 monomer (or ~9170 hexamer) molecules per cell (corresponding to ~91 μM Hfq monomer, or ~15.2 μM Hfq hexamer) under exponential growth in LB medium at 37 °C [55], i.e., a concentration that is ~5.7-fold higher than the concentration we used in our in vitro assays, indicating that Hfq-mediated protection of RNA I can occur in vivo.

As shown in Figure 1, the RNA I 5′ end deletion variant (RNA I_-8_) still binds Hfq at a concentration of 173.8 Hfq monomer (i.e., it does not completely lack binding of the chaperone; Figure 2B), indicating other site(s) binding is obviously involved. It is possible that Hfq binding to these sites is also involved in preventing RNase E cleavage. RNase E is known to have multiple cutting sites in RNA I [56], and a recent study by Richards and Belasco proposed a scanning model by which RNase E searches its downstream cleavage sites by scanning linearly from the 5′ end monophosphate along the single-stranded regions of the RNA body [57]. Obstacles, such as bound proteins or sRNAs, but not stem-loop structures between the 5′ end and the internal RNase E cleavage sites, hinder cleavage of the downstream sites [57]. Therefore, certain Hfq binding sites within the RNA I body, apart from in its 5′ end, may also interfere with the scanning activity of RNase E and cleavage of those internal RNase E sites, consequently affecting RNA I stability in vivo.

### 3.2. Hfq Binding Face for RNA I

By using Hfq proximal and distal face mutants, we have shown that antisense RNA I predominantly interacts with the proximal and not distal face of Hfq (Figure 4A,B). However, we cannot rule out the possibility that RNA I might interact with other sites of Hfq, such as the rim or C-terminal domain. A recent study using synchrotron radiation-based circular dichroism (SRCD) reported that the C-terminal domain of Hfq facilitates annealing of the first stem-loop fragment of RNA I to its perfectly complementary stem-loop fragment in RNA II [46]. Since RNA–RNA annealing is known to be catalyzed by the arginine patch on the outer rim of Hfq [17], it is possible that the rim surface of Hfq is also involved in binding to and catalyzing RNA I–RNA II annealing.

### 3.3. How Mutations of Hfq Binding Surfaces Affect RNA I Stability

A previous study has shown that degradation of RNA I_-5_ intermediates depends on 3′ polyadenylation [42], with RNA I_-5_ rendered very stable in the absence of *pcnB* (which encodes poly(A) polymerase I, PAP I), resulting in a very low plasmid copy number [42,58]. In addition, PAP I was found to co-purify with both Hfq and PNPase [59]. In our study, we detected very low amounts of RNA I_-5_ in an *hfq* deletion strain (Δ*hfq*/pBAD), slightly more in the hfq wildtype strain (MG1655/pBAD), higher again in the Hfq_WT_ overexpression strain (MG1655/pBAD-Hfq), and highest of all in the distal face Hfq_Y25D*_ mutant overexpression strain (Δ*hfq*/pBAD-Hfq_Y25D*_; Figure 5A and Figure 6A). We also showed that Hfq_Y25D*_ binds RNA I more strongly than Hfq_WT_, consistent with other proximal face-binding sRNAs (Figure 4B) [52]. This higher binding capacity of RNA I for the Hfq_Y25D*_ mutant is correlated with greater accumulation of RNA I_-5_. One possibility is that the Y25D* mutant reduces the interaction between Hfq protein and PAP I, thereby inhibiting polyadenylation and stabilizing RNA I_-5_. Moreover, the increased accumulation of RNA I_-5_, in accordance with increased expression/binding of Hfq, indicates that the presence of Hfq may impact the degree to which RNA I is polyadenylated by influencing the structure of RNA I, representing a well-known function of the chaperone protein. Although the polyadenylation activity of PAP I is reduced in an *hfq* deletion strain [59,60], our data indicated that Hfq-mediated stimulation of PAP I-driven polyadenylation might depend on the target RNA species and/or target RNA structure.

### 3.4. Possible Competition between Plasmid-Borne Antisense RNA and Chromosome-Borne sRNAs

Hfq protein is the limiting factor in sRNA-dependent regulation [61,62], so Hfq-dependent sRNAs must compete with one another for Hfq binding [53,61,62]. The competitive ability of a sRNA is affected by its Hfq binding affinity, its expression level under certain growth conditions, its secondary structure, and its Hfq binding surface preference [62,63,64]. Among these factors, the intracellular concentration of a given sRNA is considered a key driving force in replacing bound RNAs on Hfq (known as the “active cycling” model [65]), leading to rapid exchange of Hfq binding partners so that bacteria can rapidly regulate their gene expression in response to different environmental conditions by modulating expression levels of sRNAs.

Herein, we have shown that plasmid-borne antisense RNA I competes with chromosome-borne DsrA for Hfq binding in vitro, albeit with a weaker competitive ability (Figure 4C). However, antisense RNA I is expressed by a strong promoter, and its steady-state expression levels mean that it is highly abundant in bacteria [54], i.e., much more abundant than the level of endogenous sRNAs. Our in vitro Hfq–RNA binding competition assay results indicated that RNAI is much less competitive relative to DsrA, but whether RNA I is able to compete with other sRNA species for Hfq binding in vivo remains to be determined.

**Figure 7:**
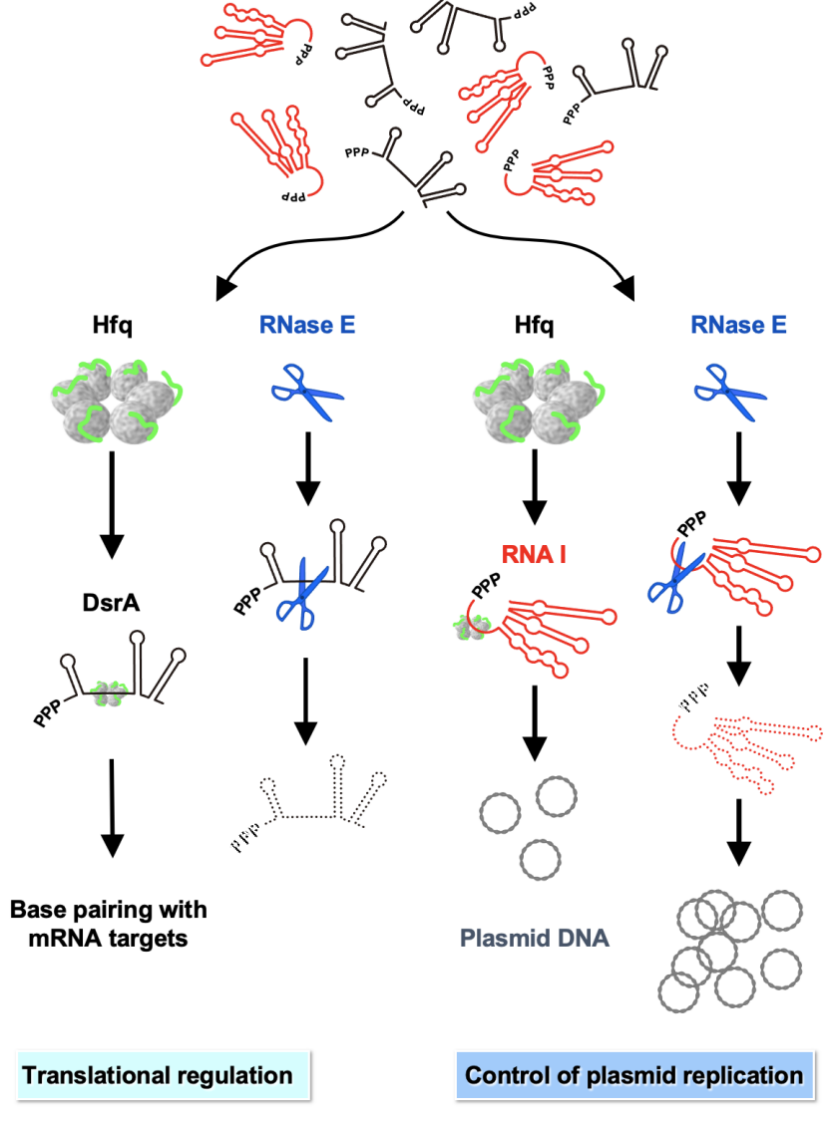
DsrA and antisense RNA I regulatory functions are controlled by Hfq binding and ribonuclease E cleavage. The left panel shows how DsrA regulation is controlled by Hfq and RNase E [21,57]. The right panel shows how RNA I regulation is controlled by Hfq and RNase E (this study). RNase E cleavage between the fifth and sixth nucleotides at the 5′ end of RNA I is the rate-limiting step in its degradation [41]. Figure 2 shows that Hfq is involved in binding to sites on RNA I other than the 5′ single-stranded region. Whether Hfq binding to this cleavage site region is actually involved in prolonging RNA I stability remains to be determined (see the Section 3 Discussion). DsrA species are shown in black. RNA I species are shown in red. Hfq binding sites and RNase E cleavage sites are shown. The Hfq protein depicted here is adapted and modified from [19]. RNA degradation is indicated as a dashed line.

In the left panel of Figure 7, we illustrate how DsrA (as an example) is protected from RNase E-mediated cleavage by binding of Hfq, thereby trans-regulating target translation [48]. In the right panel, binding of Hfq to RNA I somewhat protects the latter from RNase E-mediated degradation, which controls the ColE1 plasmid copy number (Figure 7). Overall, our study presents an example of how Hfq cooperates with RNase E in antisense RNA target regulation.

## 4. Materials and Methods

### 4.1. Bacterial Strains and Plasmid Construction

The *E. coli* k-12 bacterial wildtype MG1665 strain [66], MG1655 *hfq* deletion isogenic strain (MG1655∆*hfq*, this study), and the BL21(DE3) *rne131* strain [67] encoding a chromosomal C-terminal-truncated RNase E that retains amino acids 1 to 585 were used in our study. The BL21(DE3) *rne131* strain was used for N-RNE protein purification (see the Section 4.2 Protein Expression and Purification). MG1655 and MG1655∆*hfq* were used for Hfq protein purification or RNA I half-life studies. To generate the MG1655*∆hfq* strain, the *∆hfq-722::kan* allele (JW4130-1 strain of the Keio Collection [68]) was introduced into MG1665 by P1 transduction [69].

In order to obtain the expression plasmid of Hfq or its variants, pBAD-EBFP2 (Invitrogen, Waltham, MA, USA, pBR322 *ori*, Ap^R^) was used as the construction backbone, and all plasmids were constructed as follows. The *hfq* gene (94.8 centisomes, map position: 4,398,311 to 4,398,619, from ecocyc.org) was amplified by polymerase chain reaction (PCR) from the chromosome of the MG1655 strain. To purify Hfq protein, the *hfq* gene was inserted between the *XhoI* and *EcoRI* restriction sites of pBAD-EBFP2 to obtain a His-tagged Hfq expression plasmid (namely, pBAD-His-Hfq). To study the effect in vivo of Hfq expression on the plasmid copy number, the *hfq* gene was inserted between the *NdeI* and *EcoRI* restriction sites of pBAD-EBFP2 to obtain a non-His-tagged Hfq expression plasmid (namely, pBAD-Hfq). The primers used in this study are listed in Table 2. The restriction enzymes and T4 ligase were purchased from Thermo Fisher Scientific, Waltham, MA, USA.

To study the binding face of RNA and its effect on the plasmid copy number, Hfq point mutations were designed according to Mikulecky et al. [52]. Either pBAD-His-Hfq or pBAD-Hfq was used as the DNA template to generate the Q8A, Y25D*, K56A, H57A, and K56A/H57A *hfq* mutations by site-directed mutagenesis, using a protocol modified from a previous report [70]. The primers used to generate the respective mutations were primers 4 and 5 for the Q8A mutation, primers 6 and 7 for the Y25D* mutation, primers 8 and 9 for the K56A mutation, primers 10 and 11 for the H57A mutation, and primers 12 and 13 for the K56A/H57A double-mutation. The resulting plasmids were named: pBAD-His-Hfq _(Q8A)_, pBAD-His-Hfq _(Y25D*)_, pBAD-His-Hfq _(K56A)_, pBAD-His-Hfq _(H57A)_, pBAD-His-Hfq _(K56A/H57A)_, pBAD-Hfq _(Y25D*)_, pBAD-Hfq _(H57A)_, and pBAD-Hfq _(K56A/H57A)_. All mutant sequences were validated by DNA sequencing (conducted by the DNA Sequencing Core Facility of the Institute of Biomedical Sciences, Academia Sinica, Taipei, Taiwan). Verified plasmids were transformed into the MG1655*∆hfq* strain for further in vitro and in vivo analyses.

### 4.2. Protein Expression and Purification

For the in vitro RNase E cleavage assay, Flag-tagged N-terminal RNase E (N-RNE, encoding aa 1 to 597, carried by the pflagRE1 plasmid [71]) was purified by using the BL21(DE3) *rne131* bacterial strain as a host. The bacterial culture was grown in LB medium supplemented with 50 μg/mL of ampicillin with 160 rpm shaking at 32 °C to A_600_ = 0.5. N-RNE was induced by 1 mM IPTG for 2 h, and then the bacterial culture was harvested at 5000× *g*, 4 °C, for 5 min. The N-RNE protein was purified, as previously described [71]. To lyse bacterial cells, the bacterial culture was resuspended in Flag-binding buffer (20 mM Tris-HCl pH 8.0, 10 mM KCl, 10 mM MgCl_2_, 20 mM EDTA, 200 mM NaCl), and then passed through a French Press One-Shot Model (Constant Systems Limited, Daventry, UK) to obtain total cell lysate. Cell debris was removed by centrifuging the cell lysate (12,000 rpm, 4 °C, for 10 min) and passage through a 0.45 µm filter. The N-RNE was purified from cleared total lysate using ANTI-FLAG^®^ M2 Affinity Gel (Sigma-Aldrich, Burlington, MA, USA). Buffer A (50 mM Tris-HCl pH 8.0, 0.15 M NaCl) was used to wash non-specific proteins from the M2 affinity column, and then 1 mL of Flag-peptide (1 mg/mL) was used to elute N-RNE. The eluted N-RNE was further dialyzed to remove Flag-peptide, as described previously [71]. In brief, the purified N-RNE was placed in a dialysis bag (MWCO 6000–8000) and dialyzed twice into storage buffer (50 mM Tris-HCl pH 8.0, 0.15 M NaCl, 1 mM EDTA) for 12 h at 4 °C. The dialyzed N-RNE protein was supplemented with a final concentration of 10% glycerol and stored at −20 °C until needed.

For Hfq protein purification, the MG1655Δ*hfq* bacterial strain with either wildtype *hfq* plasmid or one of the various *hfq* mutant plasmids (pBAD-His-Hfq, pBAD-His-Hfq _(Q8A)_, pBAD-His-Hfq _(Y25D*)_, pBAD-His-Hfq _(K56A)_, pBAD-His-Hfq _(H57A)_, and pBAD-His-Hfq _(K56A/H57A)_) was cultured at 32 °C in LB medium supplemented with 50 μg/mL of ampicillin and 160 rpm shaking to A_600_ = 0.3. His-tagged Hfq and its mutant variants were induced by a final concentration of 0.1% arabinose for 2 h, as described previously [71]. Bacterial culture (500 mL equivalents) was then harvested by centrifugation and resuspended in 15 mL of His-binding buffer (20 mM sodium phosphate, 0.5 M NaCl, 40 mM imidazole) containing 1 mM of PMSF. The cells were lysed by passage through a French Press (Aminco Model FA-073) twice at 10,000 psi. Cell debris was removed, as described above. The cleared cell lysate was then incubated with 1 mL of Ni Sepharose^TM^ 6 Fast Flow (GE Healthcare, Chicago, IL, USA) for 1 h at room temperature in a Poly-Prep chromatography column (Bio-Rad, Hercules, CA, USA). The column was then washed with His-binding buffer until the pass-through buffer reached A_280_ < 0.05 mg/mL. To elute the His-tagged Hfq protein, six elutions were prepared, as follows: E1/E2 (200 mM imidazole), E3/E4 (400 mM imidazole), and E5/E6 (600 mM imidazole). His-tagged Hfq protein was eluted with 1 mL of each elution buffer (20 mM sodium phosphate, 0.5 M NaCl, plus 200 mM, 400 mM, or 600 mM imidazole). Purified His-tagged Hfq protein was concentrated using a Vivaspin 500 MWCO 3000 system (GE Healthcare) and dialyzed against PBS buffer (137 mM NaCl, 2.7 mM KCl, 8 mM Na_2_HPO_4_, 1.46 mM KH_2_PO_4_). The His tag was removed by AcTEVTM protease (Invitrogen) and confirmed by 15% SDS-PAGE. The resulting purified Hfq protein was stored at −80 °C with 5% glycerol. The purified Hfq was used to generate rabbit polyclonal antibody and for in vitro binding or competition assays.

### 4.3. RNA Preparation, N-RNE In Vitro Cleavage, In Vitro Protein–RNA Gel-Shift Assay, and RNA–Protein Competition Assay

To prepare RNA species used for N-RNE cleavage assay and gel-shift assays, RNA species were synthesized in vitro using a T7 in vitro transcription kit (MAXIscript, Ambion, Glasgow, UK) that contained purified RNA T7 RNA polymerase, according to the manufacturer’s instructions. All in-vitro-synthesized RNA species were internally labeled with [α-^32^P] UTP. In vitro RNA synthesis by T7 polymerase produces additional GGG at the 5′ end of the RNA, and it is known that this GGG does not affect RNase E cleavage on RNA I [44]. DNA templates used for in vitro transcription were generated by PCR using gene-specific primers, with the forward primers harboring the T7 promoter sequence. For full-length RNA I, pBAD plasmid was used as a template for PCR amplification with primers 14 and 15. To generate the 5′ end eight-nucleotide deletion RNA I variant (RNA I_-8_) template, primers 15 and 16 were used. To generate DsrA as a T7 in vitro transcription DNA template (DsrA on chromosome: 43.61 centisomes; map position: 2,023,337 to 2,023,251), chromosomal DNA from the MG1655 strain was used as a PCR amplification template with primers 17 and 18. After in vitro transcription, full-length ^32^P-labeled RNA transcripts were purified on a 6% polyacrylamide (19:1)/7 M urea gel and eluted in elution buffer (10 mM Tris pH 7.5, 1 mM EDTA, 0.1% SDS) at room temperature for 90 min. Eluted RNA transcripts were precipitated by adding 1 volume of isopropanol and 0.1 volume of 3 M sodium acetate (pH 7.8) and kept at −20 °C for at least 2 h to facilitate precipitation. Following precipitation, full-length RNAs were pelleted down at 13,000 rpm, 4 °C, for 15 min and washed with 70% ethanol. The RNA pellets were then re-suspended in 20 µL of DEPC H_2_O and stored at −20 °C before further experimentation. For the competition assay, non-isotope-labeled (cold) RNA transcripts were prepared, as described above, except using non-radioactive UTP and without PAGE purification. BR13 corresponding to the first 13 mers, identical to the T7 synthesized RNA I 5′ end (5′-GGG ACA GUA UUU G-3′) [44] (shown in Figure 2A) carrying the fluorescein modification at the 3′ end, was obtained from a previous study [71].

To perform in vitro Hfq protection and RNase E cleavage assays, the procedure was adapted from a previous publication [44]. In brief, 30,000 cpm (counts per minute) of purified internally ^32^P-labeled RNA I with 20 pmol of cold RNA I were pre-incubated with 0, 0.9, 1.8, or 3.6 μg of monomeric Hfq (final concentrations of 0, 4, 8, or 16 μM) based on optimized conditions in reaction buffer (20 mM Tris-HCl pH 8, 100 mM NaCl, 10 mM MgCl_2_) at 37 °C for 20 min. Then, 1 μg of N-RNE (final concentration of 750 nM) was added into each reaction for in vitro cleavage at 37 °C for 1.5 h. To stop N-RNE in vitro cleavage, 1 volume of phenol:chloroform:isoamyl alcohol (25:24:1; Affymetrix, Santa Clara, CA, USA) was added into each sample, followed by brief vortexing and centrifugation at 13,000 rpm for 2 min. The aqueous phase, which contained the RNA species, was resolved on an 8% acrylamide/7 M urea gel at 11 W for 3 h, and the radioactivity signal was visualized using a Typhoon FLA 9000 imaging system (GE Healthcare). The Hfq protection and the percentage of N-RNE cleavage were calculated by measuring and comparing the signal intensity of un-cleaved RNA I (RNA I_FL_) with the cleaved RNA I (RNA I_-5_). The ratio of RNA I_-5_ to RNA I_FL_ for each Hfq concentration was plotted using Prism (GraphPad Software version 9, Boston, MA, USA, www.graphpad.com).

To determine the binding affinity and competitive ability of RNA transcripts, we performed an in vitro protein–RNA mobility shift assay, as described previously [72]. In brief, purified sRNAs (obtained through in vitro transcription) or 5 pmol of BR13 were denatured by heating them at 90 °C for 3 min and then allowing them to refold into their natural structures by cooling them at room temperature. To determine binding affinities, an aliquot containing 30,000 cpm (counts per minute) of ^32^P-labeled RNAs was incubated with 0, 21.7, 43.4, 86.9, 173.8, 347.5, 695, 1390, 2780, or 5560 nM of monomeric Hfq protein. For the competition assay, an aliquot containing 30,000 cpm (counts per minute) of ^32^P-labeled RNA and 695 nM (determined from binding affinity data) of Hfq monomer were used. ^32^P-labeled RNA was competed by adding increasing amounts of cold DsrA (25 ng, 50 ng, 100 ng, and 200 ng). Cold RNA I ranged from 850 ng, 1.7 μg, and 3.4 μg to 6.8 μg. To measure in vitro binding of Hfq mutants to DsrA and RNA I, a concentration of either 43 nM/174 nM or 130 nM/260 nM of Hfq monomer was used, respectively. sRNAs and Hfq were incubated for 20 min at 25 °C in binding buffer (20 mM Tris-HCl pH 7.6, 0.5 M NaCl, 1 mM EDTA, 1% glycerol), before being subjected to electrophoresis on 5% polyacrylamide (19:1) native gels in 0.5 X TBE running buffer at 250 V and 4 °C for 3.5 to 4 h until the xylene cyanol dye reached the middle of the gel. After electrophoresis, gels were dried on Whatman 3 MM filter paper using a vacuum gel dryer system. Radioactivity signals were captured on a phosphor imaging plate (FUJIFILM Imaging Plate) and visualized using the Typhoon FLA 9000 imaging system (GE Healthcare). Signal intensity was quantified in the Vision Works LS system (UVP, Upland, CA, USA). The percentage of binding was obtained by measuring the total RNA signal compared to the free-form isotope-labeled RNA signals and the band-shift signals under each condition (Figure 3C). Quantifications were obtained from at least three technical repeats. Binding affinity was calculated as the Hfq concentration required for 50% binding. Plotting was performed in Prism 9 (GraphPad Software, Boston, MA, USA, www.graphpad.com). All concentrations of RNAs and protein used were determined from preliminary experiments under identical conditions.

### 4.4. RNA Half-Life Determination, Total RNA Extraction, and Northern Blot Analysis

Bacterial strains MG1655/pBAD, MG1655/pBAD-Hfq, MG1655Δ*hfq*/pBAD, MG1655Δ*hfq*/pBAD-Hfq, MG1655Δ*hfq*/pBAD-Hfq_(Y25D*)_, and MG1655Δ*hfq*/pBAD-Hfq _(K56A/H57A)_ were cultured at 37 °C with 180 rpm shaking in LB medium supplemented with an antibiotic (50 μg/mL of ampicillin). Hfq protein was induced by 0.2% arabinose at the early exponential growth phase and incubated for a further 2 to 2.5 h. Then, the bacterial samples were harvested for further RNA analysis.

To measure the RNA I half-life, de novo RNA synthesis was arrested by adding a 1/50 volume of rifampicin (50 mg/mL), and the remaining RNA I was determined by Northern blotting over time. Cultured cells were collected before and at 1, 2, 4, 6, and 8 min after rifampicin treatment, with 24 mL of cultured cells being collected at each time point and added into 4 mL of stop solution (95% EtOH, 5% saturated phenol, pH 6.6/7.9) to stop all enzymatic activities. The mixed samples were centrifuged at 4000× *g* for 10 min at 4 °C. Collected bacterial pellets were stored at −80 °C until total RNA had been prepared for half-life determination. Total RNA was extracted, as described in a previous publication [41]. Extracted RNA was separated through an 8% polyacrylamide (19:1)/7 M urea gel in 0.5 X TBE buffer using an Adjustable Height Vertical Electrophoresis System (C.B.S. Scientific, San Diego, CA, USA) at 11 W for ~3.5 h until the xylene cyanol dye had reached three-quarters the length of the gel. The RNA was transferred onto Zeta-Probe^®^ Blotting Membranes (Bio-Rad) at 400 mA, 100 min, at 4 °C in 0.5 X TBE buffer by Trans-Blot Cell (Bio-Rad) and crosslinked onto the membrane using a Stratalinker UV Crosslinker 2400 (Stratagene, San Diego, CA, USA) at a setting of 120,000 microjoules/cm^2^.

To perform Northern blot detection of RNA I, an antisense DNA probe (5′-GTA ACT GGC TTC AGC AGA GCG CAG ATA CC-3′) was labeled with [γ-^32^P] ATP at the 5′ end using T4 polynucleotide kinase (NEB). The radioactively labeled probe was purified via a MicroSpin G-25 column (GE Healthcare) before use. To detect RNA I, membranes were pre-blotted with ULTRAhyb-Oligo hybridization buffer (Ambion) for 2 h at 42 °C. The radioactively labeled probe was then added in hybridization buffer and further hybridized to the membrane at 42 °C for at least 6 h. The RNA I signal detected by the radioactive probe was visualized on an imaging plate (FUJIFILM) and detected using a Typhoon FLA 9000 imaging system (GE Healthcare). The intensity of either 5S rRNA or tRNA on the methylene blue-stained membrane was used as a loading control for normalization. Signal intensity was quantified using VisionWorks LS software version 8.20 (UVP). The RNA I half-life was determined from at least three biological repeats. The half-life of RNA I was determined from a least-squares linear fit to a semi-log plot of remaining RNA I (%) versus time.

### 4.5. Plasmid Copy Number Determination

Plasmid copy numbers were measured according to a modified protocol from a previous study [41]. In brief, 300 μL of bacterial culture, as described above for our RNA I stability determination, was first pelleted and then resuspended in 60 μL of DNA loading dye (Thermo Fisher Scientific). Then, 120 μL of phenol:chloroform:isoamyl alcohol (25:24:1; Affymetrix) was added. To disrupt the cells and extract all nucleic acid content, the phenol/chloroform mixture was vortexed through a Thermomixer comfort (Eppendorf) at 1400 rpm for 10 min at room temperature and then centrifuged at 15,000× rpm for 10 min. Twenty microliters of the resulting aqueous phase was electrophoresed on a 0.6% agarose gel in 0.5 X TAE buffer at 70 V for 90 min. Since the Hfq-overexpressing strains (i.e., MG1655/pBAD-Hfq and ∆hfq/pBAD-Hfq) have much lower plasmid copy numbers, higher amounts of DNA were loaded for quantification. DNA bands were visualized by staining the agarose gel with ethidium bromide for 5 min and UV illumination with a Gel Logic 212 PRO system (Carestream, Rochester, NY, USA). Chromosome DNA was used for normalization to obtain the relative plasmid copy number for each set of conditions, as described previously [41]. Signal intensity was measured using Fiji software version 2.3.0 [73].

### 4.6. DNA Isolation and Quantitative PCR (qPCR)

DNA isolation for further plasmid copy number determination was performed as described previously [71], with some modification. In brief, 1.5 mL of bacterial culture (grown as described above for our RNA I stability determination) was pelleted and then resuspended in 1 mL of lysis buffer (10 mM Tris-HCl pH 8.0, 1 mM EDTA pH 8.0, 0.6% SDS, 120 μg/mL proteinase K, 1 mg/mL RNase A). To lyse cells, the resuspended cell mixture was incubated at 37 °C for 1 h. DNA was further isolated with phenol:chloroform and precipitated in three volumes of EtOH at −20 °C for at least 2 h. The precipitated DNA was pelleted at 15,000 rpm, 15 min, 4 °C, and resuspended in 100 μL of TE buffer (10 mM Tris-HCl pH 8.0, 1 mM EDTA pH 8.0) for further experimental usage.

To measure the relative plasmid copy number, quantitative PCR (qPCR) was performed [74]. In brief, a 160 X dilution of isolated DNA from each strain was prepared for qPCR reaction with iQ™ SYBR^®^ Green Supermix (Bio-Rad, Hercules, CA, USA), according to the manufacturer’s instructions. qPCR was performed on a CFX Opus Real-Time PCR System (Bio-Rad, Hercules, CA, USA). Primers that generate similarly sized amplicons of either chromosome or plasmid sequences were designed: (1) for amplifying chromosome sequences to generate an 88 bp product that mapped to the *mreB* gene, or (2) for amplifying plasmid sequences to generate a 127 bp product that mapped to the *ampA* gene. Oligo primers (primers 20–23) used for qPCR (250 nM per reaction) are listed in Table 2. To obtain the relative expression of the plasmid-borne *ampA* gene representing the relative plasmid level in different strains, we adapted the 2^−ΔΔCt^ method [75]. In brief, the Ct value of the plasmid-borne *ampA* gene was first normalized against the chromosome-borne *mreB* gene to obtain ΔCt. The ΔCt value for each strain was then normalized against either MG1655/pBAD (Figure 6F) or ∆*hfq*/pBAD-Hfq_Y25D*_ (Figure 6G) to obtain ΔΔCt. Relative fold-changes of plasmid levels in different strains were calculated as 2^−ΔΔCt^. At least three biological repeats with triplicate technical repeats were performed for each target gene. Plotting was carried out in Prism 9 (GraphPad Software, Boston, MA, USA, www.graphpad.com).

### 4.7. Quantification and Statistical Analysis

The band intensity of scanned gels, membranes, or phosphor image plates was quantitated using Vision Works LS software (UVP) or Fiji software [73]. Statistical significance was determined using the unpaired Student’s *t* test. All statistical tests were performed using Prism 9 (GraphPad Software, Boston, MA, USA, www.graphpad.com).

## 5. Conclusions

Hfq is well known for its regulatory role in post-transcriptional gene regulation. In addition to *trans*-acting small RNAs, the RNA chaperone Hfq also binds *cis*- or anti-sense RNAs. In this study, we demonstrated that the Hfq binds to antisense RNA I and thereby regulates antisense RNA-mediated functions in *E. coli*. We used the classic antisense RNA-based regulatory model (i.e., RNA I) of the ColE1 plasmid and showed that Hfq binds to RNA I primarily through its proximal face, rather than the distal face. The presence of Hfq limits RNase E cleavage in vitro and prolongs RNA I stability in vivo, thereby regulating the ColE1 plasmid copy number. With limited amounts of Hfq, RNA I is able to compete for the binding to DsrA in vitro. This study adds incremental knowledge to the understanding of Hfq’s function and its role in ColE1 replication control. Because ColE1 and its derived plasmids are commonly used in modern molecular biology and DNA technology, our study provides crucial information for those using this important plasmid in studies of post-transcriptional gene regulation.

## Figures and Tables

**Table 1:** Relative binding (percentage) of RNA to mutant Hfq compared to wildtype Hfq protein at an Hfq concentration of either 174 nM (for DsrA) or 260 nM (for RNA I).

	Wildtype	Proximal Face Mutants				Distal Face Mutant
	Hfq_WT_	Q8A	K56A	H57A	K56/H57A	Y25D*
DsrA	100%	46.9 ± 4.0% ^b^	24.4 ± 4.3% ^a^	66.5 ± 17.0% ^d^	13.2 ± 6.9% ^b^	162.7 ± 13.7% ^c^
RNA I	100%	86.5 ± 3.6% ^b^	36.1 ± 5.1% ^b^	72.7 ± 5.0% ^c^	13.8 ± 8.6% ^b^	125.2 ± 22.4% ^d^

The signal intensity was quantified directly from negative film, and band intensities that are out of the linear range may imply a certain degree of error. ^a^: *p*-value ≤ 0.001; ^b^: *p*-value ≤ 0.01; ^c^: *p*-value ≤ 0.05; ^d^: *p*-value > 0.05, from an unpaired Student’s *t* test.

**Table 2:** Primers used in this study.

Primer Name	Primer Sequence	Purpose
Primer 1	5′-CCG CTC GAG AAT GGC TAA GGG GCA AT-3′	pBAD-His-Hfq construction
Primer 2	5′-CCG GAA TTC TTA TTC GGT TTC TTC GCT GTC CTG TTG-3′	pBAD-His-Hfq construction
Primer 3	5′-GGA ATT CCA TAT GGC TAA GGG GCA ATC TTT ACA-3′	pBAD-Hfq construction
Primer 4	5′-GGG CAA TCT TTA GCA GAT CCG TTC CTG AAC GCA CTG CG-3′	Hfq Q8A mutagenesis
Primer 5	5′-GTT CAG GAA CGG ATC TGC TAA AGA TTG CCC CTT AG-3′	Hfq Q8A mutagenesis
Primer 6	5′-GTT TCT ATT GAT TTG GTG AAT GGT ATT AAG CTG CAA GGG C-3′	Hfq Y25D* mutagenesis
Primer 7	5′-CCA TTC ACC AAA TCA ATA GAA ACT GGA ACA CG-3′	Hfq Y25D* mutagenesis
Primer 8	5′-GGT TTA CGC GCA CGC GAT TTC TAC TGT TGT CC-3′	Hfq K56A mutagenesis
Primer 9	5′-GAA ATC GCG TGC GCG TAA ACC ATC TGG CTG AC-3′	Hfq K56A mutagenesis
Primer 10	5′-CAG CCA GAT GGT TTA CAA GGC CGC GAT TTC TAC TGT TGT CC-3′	Hfq H57A mutagenesis
Primer 11	5′-GGA CAA CAG TAG AAA TCG CGG CCT TGT AAA CCA TCT GGC TG-3′	Hfq H57A mutagenesis
Primer 12	5′-GGT TTA CGC GGC CGC GAT TTC TAC TGT TGT CC-3′	Hfq K56A/H57A mutagenesis
Primer 13	5′-GAA ATC GCG GCC GCG TAA ACC ATC TGG CTG AC-3′	Hfq K56A/H57A mutagenesis
Primer 14	5′-TAA TAC GAC TCA CTA TAG GGA CAG TAT TTG GTA TCT GCG C-3′	RNA I_FL_ T7 template
Primer 15	5′-ACA AAA AAA CCA CCG CTA CC-3′	RNA I_FL_ T7 template
Primer 16	5′-TAA TAC GAC TCA CTA TAG GGT GGT ATC TGC GCT CTG C-3′	RNA I_-8_ T7 template
Primer 17	5′-TAA TAC GAC TCA CTA TAG GGA ACA CAT CAG ATT TCC TGG-3′	DsrA T7 template
Primer 18	5′-AAA TCC CGA CCC TGA GG-3′	DsrA T7 template
Primer 19	5′-GTA ACT GGC TTC AGC AGA GCG CAG ATA CC-3′	RNA I oligo probe
Primer 20	CGG TTC TAT GGT GGT TGA	qPCR primer for *mreB*
Primer 21	GCG CAC AGA AGA GGA GTA	qPCR primer for *mreB*
Primer 22	TTC CGG CTG GCT GGT TTA TT	qPCR primer for *ampA*
Primer 23	TGA CTC CCC GTC GTG TAG AT	qPCR primer for *ampA*

Underlined sequence in primer 1: *XhoI* restriction site; primer 2: *EcoRI* restriction site; primer 3: *NdeI* restriction site; primers 4 to 13: mutated codons; primers 14, 16, and 17: T7 promoter.

## Data Availability

Data is contained within the article.
